# Evidence for the involvement of cofilin in *Aspergillus fumigatus* internalization into type II alveolar epithelial cells

**DOI:** 10.1186/s12866-015-0500-y

**Published:** 2015-08-13

**Authors:** Zhiyao Bao, Xuelin Han, Fangyan Chen, Xiaodong Jia, Jingya Zhao, Changjian Zhang, Chen Yong, Shuguang Tian, Xin Zhou, Li Han

**Affiliations:** Department of Respiratory Medicine, Shanghai first people’s hospital, Shanghai Jiao Tong University, No. 100, Haining Road, Shanghai, 200080 China; Department for Hospital Infection Control & Research, Institute of Disease Control & Prevention of PLA, Academy of Military Medical Sciences, Fengtai Dong Street 20, Beijing, 100071 China; Department of Respiratory Medicine, Ruijin hospital, School of Medicine, Shanghai Jiaotong University, No. 197 Ruijin Er Road, Shanghai, 200025 China

**Keywords:** *Aspergillus fumigatus*, Internalization, Cofilin, Lung epithelial cell

## Abstract

**Background:**

The internalization of *Aspergillus fumigatus* into alveolar epithelial cells (AECs) is tightly controlled by host cellular actin dynamics, which require close modulation of the ADF (actin depolymerizing factor)/cofilin family. However, the role of cofilin in *A. fumigatus* internalization into AECs remains unclear.

**Results:**

Here, we demonstrated that germinated *A. fumigatus* conidia were able to induce phosphorylation of cofilin in A549 cells during the early stage of internalization. The modulation of cofilin activity by overexpression, knockdown, or mutation of the cofilin gene in A549 cells decreased the efficacy of *A. fumigatus* internalization. Reducing the phosphorylation status of cofilin with BMS-5 (LIM kinase inhibitor) or overexpression of the slingshot phosphatases also impeded *A. fumigatus* internalization. Both the *C. botulimun* C3 transferase (a specific RhoA inhibitor) and Y27632 (a specific ROCK inhibitor) reduced the internalization of *A. fumigatus* and the level of phosphorylated cofilin. β-1,3-glucan (the major component of the conidial cell wall) and its host cell receptor dectin-1 did not seem to be associated with cofilin phosphorylation during *A. fumigatus* infection.

**Conclusion:**

These results indicated that cofilin might be involved in the modulation of *A. fumigatus* internalization into type II alveolar epithelial cells through the RhoA-ROCK-LIM kinase pathway.

## Background

The opportunistic pathogen *Aspergillus fumigatus* is a saprophytic filamentous fungus that causes a wide range of diseases, including allergic bronchopulmonary aspergillosis, aspergilloma and invasive aspergillosis. It propagates through airborne conidia (spores) that are inhaled into the small airways, where they may germinate and initiate an infection. Alveolar epithelial cells not only act as an anatomic barrier to defend against *A. fumigatus*, but can also be invaded by conidia, thereby serving as reservoirs for evasion from immune attack and dissemination throughout the host [1]. Similar to other modes of cytokinesis (*e.g.*, cell division and migration), the internalization of *A. fumigatus* into epithelial cells has been reported to be dependent on the dynamic assembly of the actin cytoskeleton, which induces the invagination of the host cell membrane and engulfs the conidia using pseudopods [2, 3].

The dynamic processes of the actin cytoskeleton have been proposed to be highly regulated by various factors, among which the ADF (actin depolymerizing factor)/cofilin family plays an essential and conserved role [4]. In mammalian cells, the ADF/cofilin family consists of three similar members: cofilin-1, cofilin-2 (distributed specifically in muscle cells) and ADF (destrin) [5, 6]. Cofilin-1 is the most ubiquitous form and has been the most widely studied. Herein, we focus on cofilin-1 and refer to it as ‘cofilin’. Cofilin binds the minus end of actin and inhibits the formation of actin filaments (F-actin), whereas the Arp2/3 protein binds to the plus end of actin and activates the formation of F-actin [7, 8]. When the third amino acid of the conserved N-terminus (Ser) is phosphorylated, cofilin loses its actin depolymerizing activity, leading to the inhibition of F-actin severing and the production of filopodia/lamellipodia. The threonine kinase family LIM kinases (LIMK) phosphorylate and deactivate cofilin. Accordingly, dephosphorylation by the slingshot phosphatases (SSH) results in reactivation of the actin binding activity of cofilin [9]. The LIMK are activated by phosphorylation through divergent Rho GTPase pathways: Rac/Cdc42 acts through p21-activated kinase (PAK) 1 and PAK4, while RhoA (Ras homologue gene family, member A) acts through ROCK (Rho-associated coiled-coil-containing kinase) [10, 11].

Recent studies have shown that cofilin activity is required for entry into host cells by many pathogens, including HIV (human immunodeficiency virus), *Cryptococcus neoformans*, and *Listeria monocytogenes* [12–14]. However, the expression, distribution and phosphorylation cycle of cofilin during the process of invasion is specific to the pathogens, host cells and involved receptors. HIV virus-induced cofilin activation is mediated by the gp120-triggered transient activation of LIMK. Knockdown of LIMK through siRNA decreases filamentous actin, increases CXCR4 trafficking, and diminishes viral DNA synthesis [12, 15]. Chen and colleagues demonstrated that the dephosphorylated form of cofilin was increased during cryptococcal adherence to human brain microvascular endothelial cells concomitant with actin rearrangement through the ROCK-LIMK-cofilin pathway [13]. Our previous study showed that the internalization of *Listeria monocytogenes* into Vero cells was tightly controlled by the phospho-cycling of cofilin, which mediated PLD1 activation during the internalization procedure [14]. Moreover, host cell PLD activity induced by β-1,3-glucan on the surface of the swollen conidia was important for the efficient internalization of *A. fumigatus* into A549 cells [16]. Due to the vital role of cofilin in the invasion process of host cells by pathogens, investigating the involvement and function of cofilin in host cells during *A. fumigatus* infection is of considerable importance.

In the present study, we demonstrated that cofilin was involved in the internalization of *A. fumigatus* into AECs through its phosphorylation cycle. Moreover, we showed that the RhoA-ROCK-LIMK pathway acted as an upstream regulator to control cofilin activity during *A. fumigatus* internalization.

## Methods

### Cell line and A. fumigatus strain

The type II human alveolar epithelia cell line A549 was obtained from ATCC (America Type

Culture Collection) and cultured in DMEM (GIBCO) supplemented with 10 % foetal calf serum, 100 U/mL streptomycin, and 100 U/mL penicillin at 37 °C in an incubator with a humidified atmosphere of 5 % CO_2_ and 95 % room-air. *A. fumigatus* ATCC13073 constitutively expressing green fluorescent protein and *A. fumigatus ΔrodA* (from *A. fumigatus* CEA17*ku80Δ*) that has lost the hydrophobic layer were used in this study. The fungi were propagated on Sabouraud dextrose agar (10 g/L peptone, 40 g/L glucose, and 20 g/L agar) for 5–8 days at 37 °C. After 5–8 days of culture, *A. fumigatus* conidia were dislodged from the agar plates by gentle washing and resuspended in sterile phosphate-buffered saline supplemented with 0.1 % Tween-20 (PBST). Then, the conidia were passed through 8 layers of sterile gauze to remove the hyphal fragments and enumerated on a haemacytometer. The conidia were incubated at 37 °C in liquid Sabouraud media and shaken at 200 rpm for 4 h to obtain swollen conidia; then, they were washed twice with PBST and stored at 4 °C for use within 24 h.

### Construction of the rodA mutant strain

**Fig. 1:**
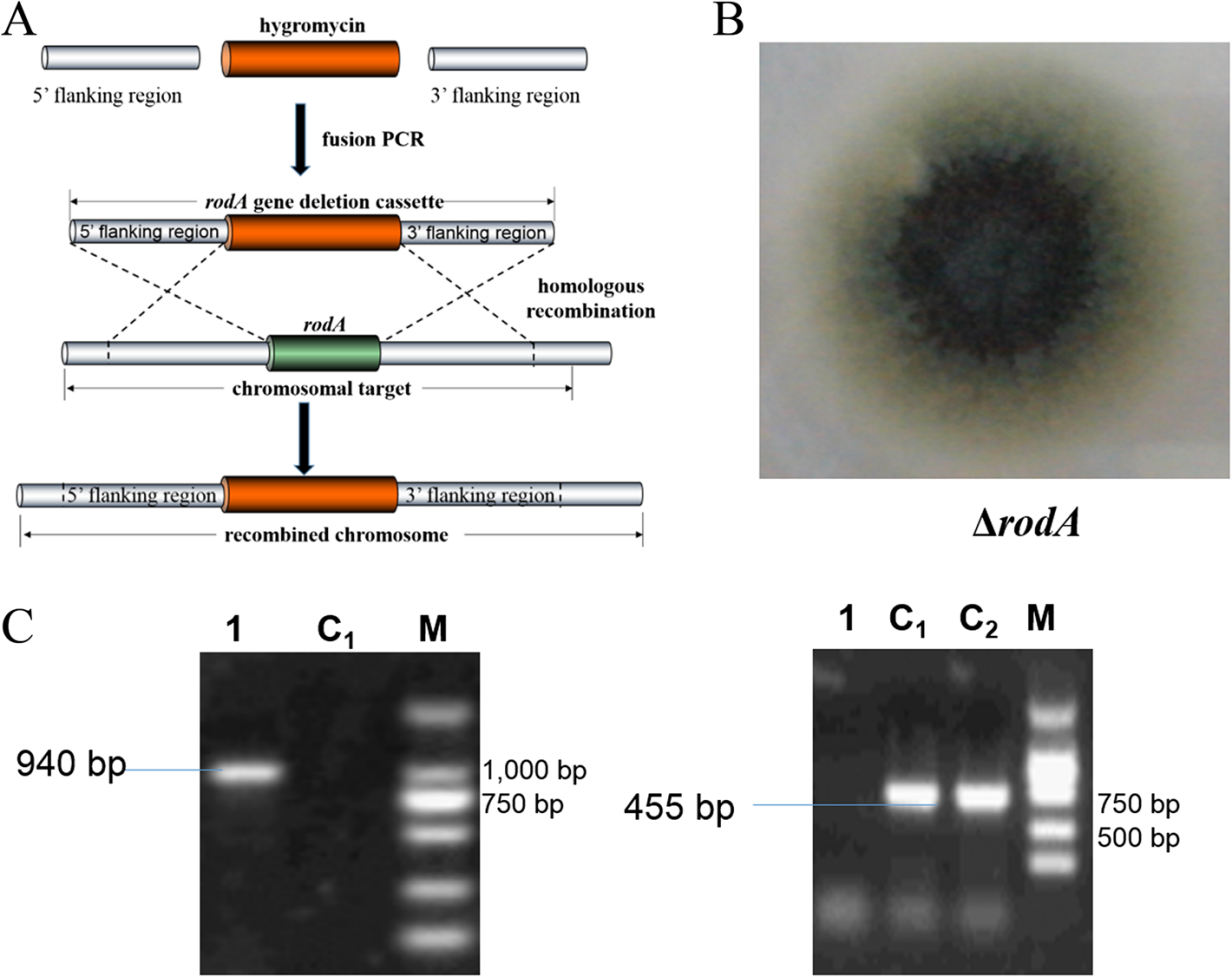
Schematic diagram and verification of the *ΔrodA* strain. **a** Strategy of construction of Δ*rodA* based on fusion PCR and homologous recombination. **b** Morphology of Δ*rodA* mutant strain on AMM including 1.2 M sorbitol and 200 μg/ml hygromycin. (C) PCR validation of Δ*rodA* mutant strain. In left panel, the PCR amplified a 0.94 kb fragment between 5’ flanking region and hygromycin fragment in Δ*rodA* mutant strain (Lane 1) and nothing in the wild type strain (Lane C1). In right panel, the PCR amplified a 0.455 kb fragment of *rodA* in the wild type strain (Lane C1 and C2) and nothing in Δ*rodA* mutant strain (Lane 1)

Oligonucleotides used in this study can be found in Table 1. The Δ*rodA* mutant was generated as described previously [17] using a method based on homologous recombination. Briefly, the flanking fragments of the *rodA* gene (approximately 1 kb each) were amplified by PCR from chromosomal DNA of non-homologous end-joining-deficient strain CEA17*ku80Δ*. And the hygromycin resistance fragment was amplified from pDHt-hph-hindIII-sacI. The *rodA* deletion cassette including two flanking fragments and a hygromycin fragment was constructed by fusion PCR and purified for transformation. *A. fumigatus* protoplasts were generated by Lysing Enzymes (Sigma, L1412). The *rodA* deletion cassette was transformed to protoplasts in the presence of polyethylene glycol (PEG). The transformants were screened on AMM plates containing 1.2 M sorbitol and 200 μg/ml hygromycin (Fig. 1).

**Table 1:** Oligonucleotides used in this study

Name	Sequence
rod-upS	GACTAGTGCACGAGGAGGCTTCTTATT
rod-upA	GAGCTCCAGCTTTTGTTCCTCACGGTGATGTCGTCAGG
rod-dwS	AAGCTTATCGATACCGTCGACGGTTCCCTCATTGGACTGG
rod-dwA	CCGCTCGAGGCAACAACATCAGCACCCT
hph-rodS	CCTGACGACATCACCGTGAGGAACAAAAGCTGGAGCTC
hph-rodA	CCAGTCCAATGAGGGAACCGTCGACGGTATCGATAAGCTT
rod-yS	GGCTGCGAGATTGCGATAA
rod-yA	CGATATTTGGACGCCCTAC
rod-rodS	GCTGCTGGCAATGGTGTT
rod-rodA	CCGCTCTGATGGACGTTAGT

### Chemical reagents, antibodies, siRNA and plasmids

BMS-5 from Synkinase (Australian, SYN-1024) was dissolved in dimethyl sulfoxide (DMSO) and Y27632 from Sigma-Aldrich (USA, Y0503) was dissolved in sterile double-distilled water for use as specific inhibitors in the experiments. β-1,3-glucan from *Euglena gracilis* (Flucan 89862) was dissolved in PBS, and a fresh stock solution was prepared for each *in vitro* experiment. For the western blot analysis, the rabbit-monoclonal anti-phospho-cofilin (3313) antibody was purchased from Cell Signalling Technology (USA). The mouse monoclonal anti-β-actin (sc-47778) and rabbit-polyclonal anti-cofilin (sc-33779) antibodies were obtained from Santa Cruz Biotechnology (USA). The HPR-conjugated goat anti-mouse IgG and HRP-conjugated goat anti-rabbit IgG antibodies were obtained from ZSGB-BIO (China). For the immunofluorescence analysis, we used the same anti-phospho-cofilin antibody listed above; the TRITC-conjugated goat anti-rabbit IgG secondary antibody was also obtained from ZSGB-BIO (China). For blocking analysis, we used the anti-dectin-1 monoclonal antibody GE2 (ab82888) that was commercially available from Abcam Inc.; the isotype control monoclonal antibody (IgG1) was purchased from eBioscience. For cofilin interference, pairs of 21-nucleotide sense and antisense RNA oligomers were designed and chemically synthesized by Sigma-Aldrich. The oligonucleotides for cofilin were as follows: sense, 5’-GAA GGA GGA UCU GGU GUU UdTdT-3’ and antisense, 5’-AAA CAC CAG AUC CUC CUU CdTdT-3’ (SASI_ Hs01_00078353). The siRNA universal negative control #1 (Sigma) was used. Wild-type cofilin and the un-phosphorylatable S3A cofilin (each subcloned into pcDL-SRα), slingshot phosphatase 1 L (subcloned into pCDNA3.1/myc-tagged), and C3T (C3 transferase, subcloned into pEF/myc-tagged) plasmids were preserved in our laboratory.

### Transfection

The transfection of A549 cells with plasmids or siRNAs was performed with Lipofectamine 2000 (USA, Invitrogen). For transfection of 50 ml culture flasks, 10 μg of plasmid DNA or 250 pmol of siRNA was diluted in 500 μl of Opti-MEM I reduced-serum medium (USA, GIBCO) and thoroughly mixed with 20 μl of Lipofectamine 2000 diluted in 500 μl of Opti-MEM I reduced-serum medium and incubated for 20 min. The transfection mixture was added to each flask containing A549 cells (70 %-80 % confluence). At 5 h post-transfection, the mixture was replaced with growth medium. At 36 h post-transfection, the cells were infected with *A. fumigatus* for further experiments. Expression of the encoded proteins was verified by western blotting of the cell lysates with specific antibodies.

### Analysis of A. fumigatus internalization

The nystatin protection method was used to investigate the internalization of *A. fumigatus* into A549 cells. A549 cells transfected with different plasmids or siRNAs were seeded 20 ~ 24 h prior to the experiment at a density of 1×10^4^ cells/well in 96-well plates (Corning). Normal A549 cells were seeded in the same way, and then pretreated with different inhibitors at 37 °C for 1 h in 5 % CO_2_. The above cells were incubated with swollen conidia of *A. fumigatus* ATCC13073 at a multiplicity of infection (MOI) of 10 for 1 h at 37 °C in 5 % CO_2_ with growth medium. Then, the cells were washed 3 times with PBST and incubated with nystatin (100 μl/well of a 20 μg/mL concentration) in DMEM for 4 h at 37 °C. Subsequently, the monolayers were washed three times with PBST and lysed by incubating in 0.25 % Triton X-100 diluted in PBS for 15 min. The released conidia were diluted and plated onto SDA agar (3 replicate plates/well) and incubated at 37 °C for 18 h. The colonies were counted to enumerate the total intracellular conidia. The internalization capacity is expressed as a percentage of the initial inoculum.

### Western blotting

Cells were lysed for 30 min in cold lysis buffer containing 150 μM NaCl, 5 % (w/v) 1 M Tris–HCl (pH 7.5), 0.5 μM EDTA•Na_2_, and 1 % (w/v) NP_4_O. After centrifugation at 13,000 rpm for 20 min, the supernatants were collected and served as the total cellular protein extracts. Equal amounts of protein were separated by SDS-PAGE (5 % to 12 % gradient) and transferred to a PVDF membrane. Then, the membrane was incubated for 2 h with the indicated primary antibodies and subsequently incubated for 1 h with the HRP-conjugated secondary antibody. The proteins were visualized by enhanced chemiluminescence (USA, Santa Cruz Biotechnology). Image J software was used to quantify the band intensity. The signal intensity for each protein was normalized to the signal intensity measured for β-actin and was expressed as the fold increase relative to the control.

### Immunofluorescence staining

A549 cells (1*10^5^/well) were seeded onto coverslips in 24-well plates and grown for 24 h for immunofluorescence analysis. Swollen conidia of *A. fumigatus* at an MOI of 10 were added to the culture plate wells for infection for 30 min. The cells were washed with ice-cold PBS and fixed in ice-cold 4 % paraformaldehyde/PBS to stop the internalization process for 20 min at 4 °C. Then, the cells were permeabilized for 5 min at room temperature with 0.05 % TritonX-100 in PBS buffer. The samples were blocked in 5 % bovine serum albumin (BSA). The primary antibody used for cofilin/ p-cofilin staining was anti-cofilin (sc33779, 1:50)/anti-phosphate-cofilin (3313, 1:50) with incubation overnight at 4 °C; the secondary antibody used for cofilin/p-cofilin was TRITC-conjugated goat anti-rabbit IgG (1:50) with incubation at room temperature for 1 h. Additionally, 1 mg/ml DAPI (4’,6’ diamidino-2-phenylindole) was added for 5 min to label the nuclei. The preparations were observed under a BX51 fluorescent microscope (Olympus 400×), and an Olympus DP71 camera using Image Pro Express 6.0 (Media Cybernetics Inc., MA, USA) was used to process the images and detect colocalization between *A. fumigatus* and cellular p-cofilin.

### Antibody inhibition experiments

To block the dectin-1 receptor, A549 cells were incubated in growth medium containing 5 μg/ml anti-dectin-1 monoclonal antibody GE2 or the isotype control antibody for 30 min. Then, the swollen conidia were added to the cells at an MOI of 10 for 30 min prior to western blot analysis.

### Statistical analysis

Data shown in the figures were either representative experiments or represented as the means ± SE of 3 independent experiments. Means were statistically compared using either Student’s unpaired *t*-test or the one-way analysis of variance test. The difference was regarded as significant with *P* < 0.05.

## Results

### Change in cofilin phosphorylation during A. fumigatus internalization into A549 cells

**Fig. 2:**
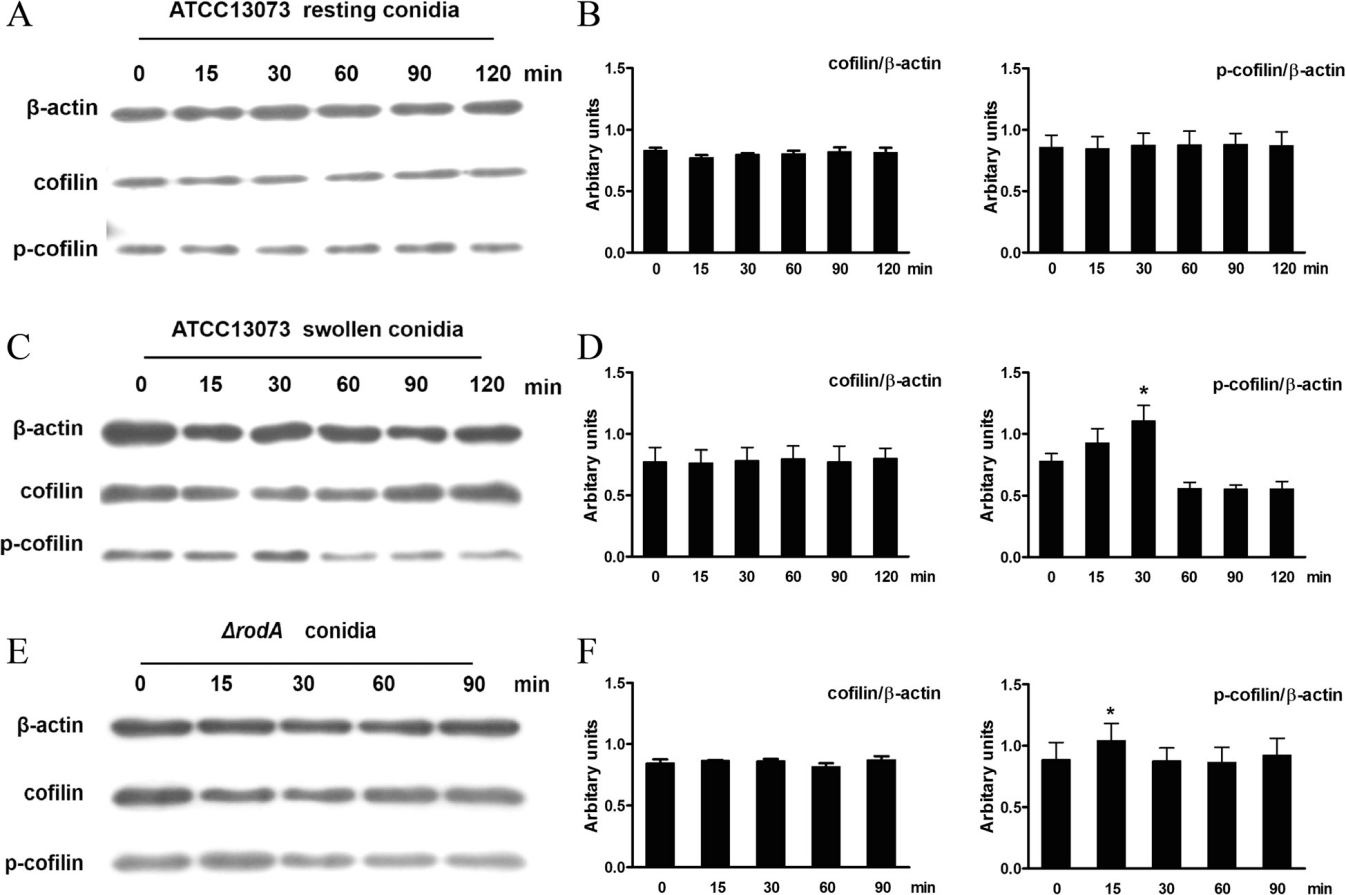
*A. fumigatus* infection induced a change in cofilin phosphorylation. A549 cells were infected with the resting conidia of *A. fumigatus* ATCC13073 (**a, b**), swollen conidia of *A. fumigatus* ATCC13073 (**c, d**) or *ΔrodA*-mutant conidia (**e**,** f**) at an MOI of 10 for the indicated times. Cofilin and p-cofilin were measured by western blotting with the indicated antibodies (**a**, **c**, and **e**); the densitometric analyses of the western blots for three independent experiments are shown (**b**, **d**, and **f**). The blots are characteristic of three independent experiments and data are shown as the mean ± SE, * *P* < 0.05 compared with 0

**Fig. 3:**
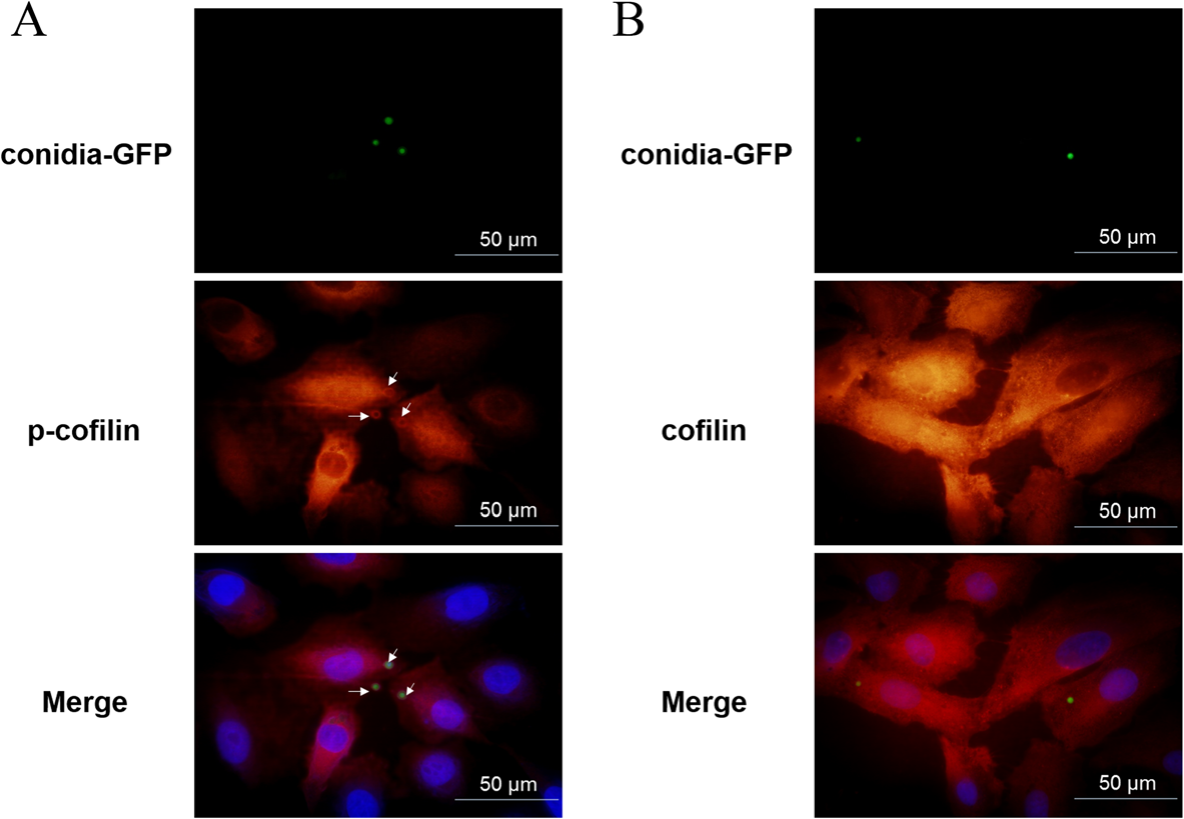
The internalized swollen conidia were surrounded by the increased p-cofilin. **a** Internalization of swollen conidia of *A. fumigatus* ATCC13073 into A549 cells (MOI = 10) was monitored at 30 mpi by fluorescence microscopy (green, *A. fumigatus* conidia; red, p-cofilin; blue, nuclei). **b** Internalization of swollen conidia of *A. fumigatus* ATCC13073 into A549 cells (MOI = 10) was monitored at 30 mpi by fluorescence microscopy (green, *A. fumigatus* conidia; red, cofilin; blue, nuclei). The images were processed with Image Pro Express 6.0, and the merged fluorescence images are shown. The data represent three similar experiments, and the arrows (white) indicate the co-localization of p-cofilin and conidia. Scale bar, 50 μm

Similar to many microbial pathogens (*i.e.*, Herpes simplex virus 1 (HSV-1) [18] and *Bartonella henselae* [19]), *A. fumigatus* induced visible alterations in the host actin cytoskeleton during internalization into nonprofessional phagocytic cells [20]. Because cofilin is an important modulator of host actin dynamics, its involvement in *A. fumigatus* internalization into A549 cells was investigated. As determined by western blot analysis, resting conidia of *A. fumigatus* ATCC13073 did not induce significant alterations in the total cofilin protein levels and phosphorylated-cofilin (p-cofilin) levels during the interaction with A549 cells for 2 h (Fig. 2a and b). In contrast, the germinated swollen conidia induced an obvious increase in p-cofilin during the early stage of the interaction with A549 cells, but had no effect on the total cofilin protein level. The level of p-cofilin reached its peak at 30 mpi (minute post infection), and then returned to the baseline (Fig. 2c and d). Because the germination of the *A. fumigatus* conidia was accompanied by the loss of the hydrophobic layer from the resting conidia, we investigated whether conidia without the hydrophobic layer could induce cofilin phosphorylation during internalization. Using a homologous recombination technique, the *rodA* gene (a major gene encoding a factor involved in hydrophobic layer formation) was deleted and the *ΔrodA* mutant of *A. fumigatus* was generated. The *ΔrodA-*mutant conidia obviously induced cofilin phosphorylation at approximately 15 mpi (which was earlier than the swollen conidia of wild-type *A. fumigatus;* Fig. 2e and f), but did not affect the level of total cofilin protein. Finally, we investigated the distribution of cofilin/p-cofilin during *A. fumigatus* swollen conidia internalization into A549 cells by immunofluorescence staining to support the western blotting results. As illustrated in Fig. 3a, the green-fluorescent-labelled swollen conidia of *A. fumigatus* 13073 were surrounded with red-labelled p-cofilin in A549 cells when the swollen conidia were incubated with cells for 30 mpi; however, cofilin did not surround the internalized swollen conidia (Fig. 3b). Taken together, our results suggest that *A. fumigatus* swollen conidia is able to induce an alteration in cofilin phosphorylation in AECs and an increase in the colocalization of p-cofilin with the swollen conidia during the early stage of *A. fumigatus* internalization into A549 cells.

### Modulation of cofilin expression affected A. fumigatus internalization

**Fig. 4:**
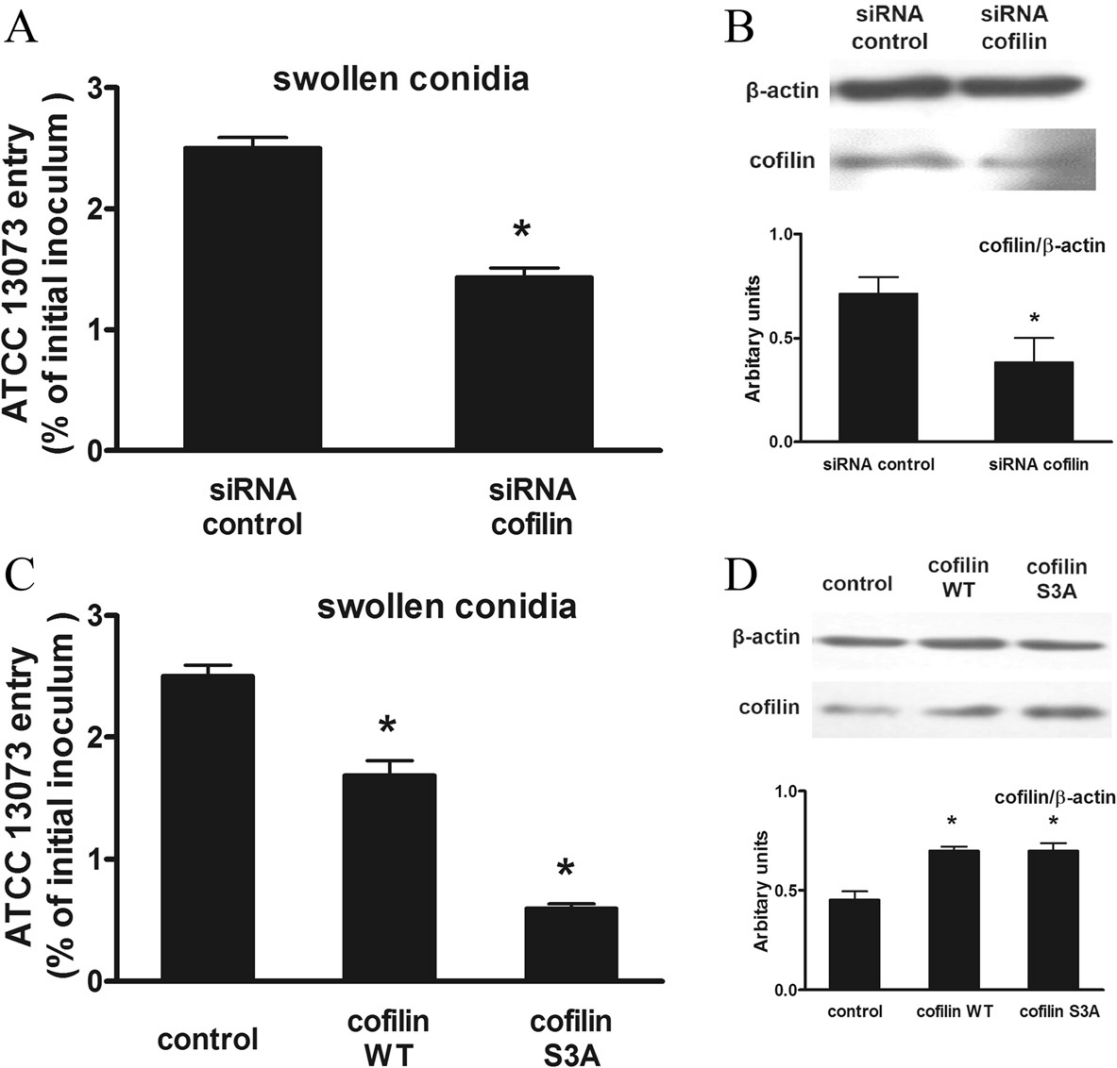
Modulation of cofilin expression affected *A. fumigatus* internalization. A549 cells were transfected with siRNA control, cofilin-specific siRNA (**a, b**) an empty vector (control), or pcDL-SRα encoding wild-type cofilin or unphosphorylatable S3A cofilin (**c** **, d**). After 36 h, cells were infected with the swollen conidia at an MOI of 10 for 1 h. *A. fumigatus* internalization was analysed by the nystatin protection assay (**a**, **c**). The expression of the indicated protein was detected by western blotting. The densitometric analyses of western blots from three independent experiments are shown (**b, d**). Mean ± SE from 3 independent experiments are shown, *P < 0.05 compared with the control

To further confirm the role of cofilin in *A. fumigatus* internalization, A549 cells were infected with swollen conidia after transient transfection with a cofilin-specific small interfering RNA (siRNA) or plasmids. The internalization process of the swollen conidia was clearly impeded by the cofilin-specific siRNA (Fig. 4a). The cofilin siRNA effectively reduced the expression of the cofilin protein detected by western blotting (Fig. 4b). Overexpression of wild-type cofilin (cofilin WT) has been reported to increase cofilin activity, and the expression of a mutant carrying the S3A substitution has also been successfully used to mimic active cofilin [21]. Interestingly, the expression of both cofilin plasmids inhibited the internalization of the swollen conidia, similar to the inhibition by the siRNA targeting cofilin (Fig. 4c). These data implied that both down- and up-regulation of cofilin activity inhibited the internalization of *A. fumigatus* swollen conidia.

### The proper phosphorylation status of cofilin was required for efficient internalization of A. fumigatus

**Fig. 5:**
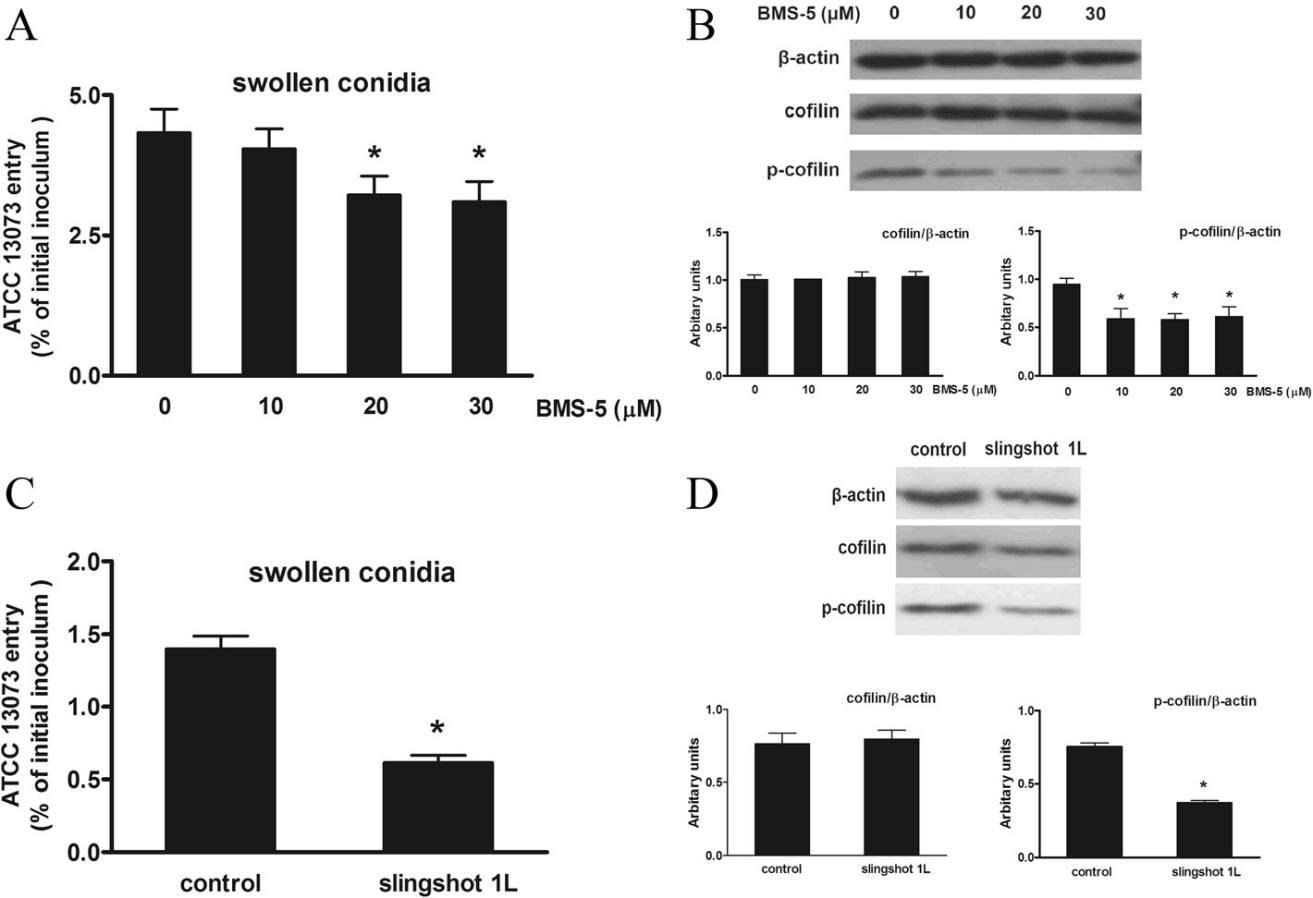
LIMK and SSH modulated the phosphorylation cycle of cofilin to affect the internalization of *A. fumigatus* swollen conidia. A549 cells were either treated with BMS-5 (the pharmacological inhibitor of LIMK) at the indicated doses for 1 h or transfected with pCDNA3.1/myc-tagged-SSH1L or the empty vector (control) for 36 h prior to inoculation with *A. fumigatus*. The cells were inoculated with swollen conidia for 1 h at an MOI of 10; then, the internalization and protein levels were analysed. *A. fumigatus* internalization was analysed by the nystatin protection assay (**a**, **c**). The expression of cofilin and p-cofilin were measured by western blotting with the indicated antibodies. The densitometric analyses of the western blots are shown (**b**,** d**). The means ± SE of three independent experiments are shown. * *P* < 0.05 compared with 0 or the control. Blots are characteristic of three independent experiments

The cofilin phosphorylation cycle is controlled by the opposing actions of LIMK and the SSH phosphatase. Next, we sought to identify the role of the cofilin phosphorylation status in *A. fumigatus* invasion of A549 cells. Initially, we inhibited the activity of LIMK with its pharmacological inhibitor BMS-5 [22]. The results showed that BMS-5 inhibited the internalization of *A. fumigatus* swollen conidia in a dose-dependent manner (Fig. 5a) and reduced the p-cofilin level in infected A549 cell lysates (Fig. 5b). We also observed an effect on *A. fumigatus* internalization by the SSH phosphatase. The overexpression of slingshot phosphatase 1 L was used to mimic the increase in SSH activity. The increased activity of SSH in A549 cells impeded the internalization of the swollen conidia and decreased the level of p-cofilin (Fig. 5c and d). The data above established that the proper phosphorylation status of cofilin should be maintained to ensure the efficient internalization of swollen conidia into A549 cells.

### Involvement of RhoA and ROCK in A. fumigatus internalization and cofilin phosphorylation

**Fig. 6:**
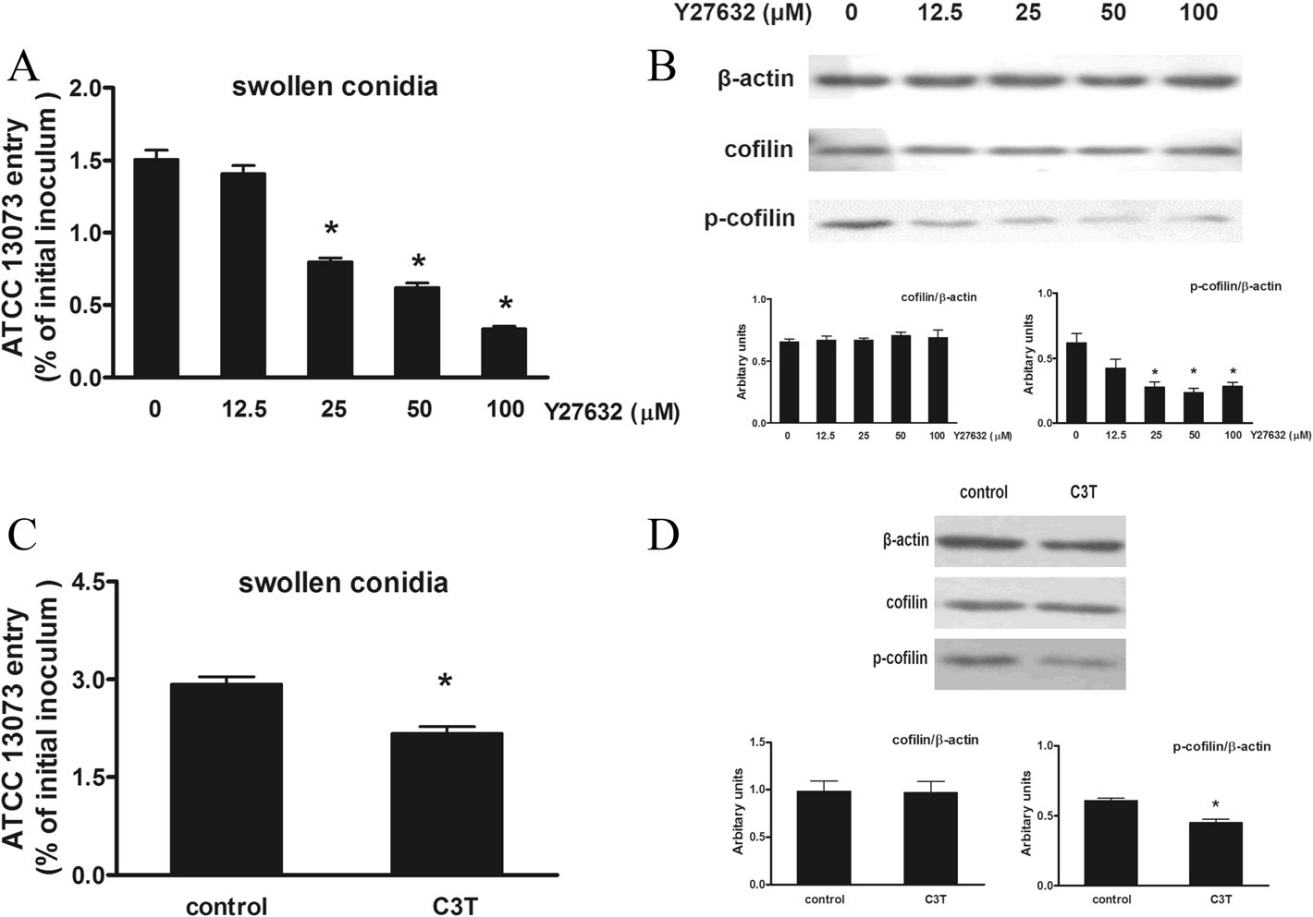
ROCK and RhoA were involved in the regulation of *A. fumigatus* invasion and cofilin phosphorylation. A549 cells were treated with Y27632 (a specific inhibitor of ROCK) at the indicated concentration for 1 h (**a**, **b**) or transfected with pEF-C3T or the empty vector (control) for 36 h (**c**, **d**) prior to inoculation with *A. fumigatus*. The cells were inoculated with swollen conidia for 1 h at an MOI of 10. Then, *A. fumigatus* internalization was analysed by the nystatin protection assay (**a**, **c**). The expression of cofilin and p-cofilin as measured by western blotting with the indicated antibodies. The densitometric analyses of the western blots are shown (B, D). The means ± SE of three independent experiments are shown. *P < 0.05 compared with 0 or control. Blots are characteristic of three independent experiments

ROCK is a downstream effector of Rho that does not phosphorylate cofilin directly. However, ROCK phosphorylates LIMK, which in turn converts cofilin into the phosphorylated form [11, 23]. Therefore, we tested whether the RhoA/ROCK signal pathway was involved in *A. fumigatus* internalization and thus the regulation of cofilin phosphorylation. Significantly, Y27632 (a specific inhibitor of ROCK) impeded the internalization of the swollen conidia of *A. fumigatus* and cofilin phosphorylation in a dose-dependent manner (Fig. 6a and b). Moreover, the expression of C3T (an inhibitor of RhoA) reduced the internalization of the swollen conidia and the p-cofilin level (Fig. 6c and d). Therefore, *A. fumigatus*-induced cofilin phosphorylation and internalization of *A. fumigatus* might be partially modulated through the RhoA/ROCK signalling pathway.

### β-1,3-glucan signalling was not associated with cofilin phosphorylation induced by A. fumigatus

**Fig. 7:**
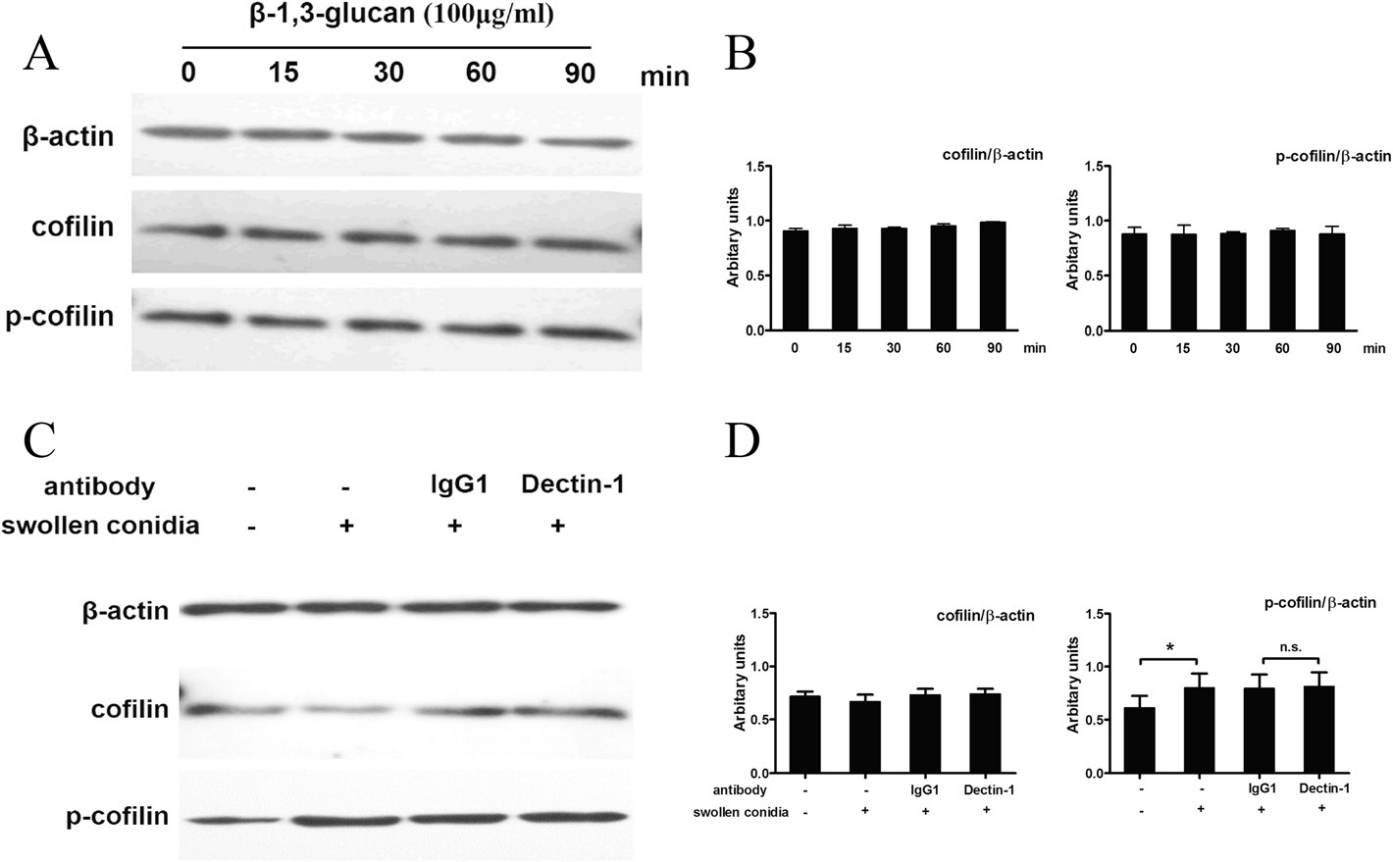
β-1,3-glucan signalling was not associated with the cofilin phosphorylation induced by *A. fumigatus.* A549 cells were stimulated with the indicated concentrations of β-1,3-glucan (100 ug/ml) for the indicated times. Thereafter, the level of cofilin and p-cofilin were detected by western blotting (**a**). Other A549 cells were first incubated with complete DMEM media, complete DMEM supplemented with 5 μg/ml of isotype control antibody (IgG1) or complete DMEM supplemented with 5 μg/ml of anti-dectin-1 mAb (ab82888) for 30 min. Then, they were infected with the swollen conidia of *A. fumigatus* 13073 at an MOI of 10 for 30 min. Finally expression of cofilin/p-cofilin was analysed by western blotting (**c**). The densitometric analyses of the western blots for three independent experiments are shown (**b**,** d**). * *P* < 0.05, and n.s. represent no difference between the columns. Blots are characteristic of three independent experiments

*A. fumigatus* conidia swelling was accompanied by surface stage-specific exposure of β-1,3-glucan polymers [24]. Our previous studies showed that β-1,3-glucan could induce the internalization of the swollen conidia of *A. fumigatus* [16]. Here, we investigated the possible role of β-1,3-glucan in cofilin phosphorylation. A549 cells were stimulated at the indicated time points with two concentrations of β-1,3-glucan polymers: 50 μg/ml (data not shown) and 100 μg/ml. As shown in Fig. 7a, β-1,3-glucan did not induce the change in cofilin phosphorylation during 90 min of incubation. Because dectin-1 is the major receptor for β-1,3-glucan, we used the monoclonal GE2 antibody against dectin-1 to block the dectin-1 receptor. As shown in Fig. 7c, cofilin phosphorylation induced by swollen conidia of *A. fumigatus* was not reduced by pretreatment with the anti-dectin-1 antibody; indeed, the results were similar to those obtained following pretreatment with the IgG isotype control antibody. Taken together, these results indicated that the cofilin phosphorylation induced by *A. fumigatus* internalization into A549 might not be initiated by β-1,3-glucan signalling.

## Discussion

Although actin reorganization during the entry of microbial pathogens has been reported in several other cell lines [25], the mechanism underlying this phenomenon has remained largely uncertain, especially with regard to the internalization of *A. fumigatus* into non-phagocytic cells. Here, we demonstrated for the first time an inter-relationship between the phosphorylation of cofilin and the internalization of swollen conidia of *A. fumigatus* into alveolar epithelial cells. In contrast, resting conidia could not induce cofilin phosphorylation. This result is in accordance with a previous study showing that swollen conidia, but not resting conidia, are able to induce IL-8 production in respiratory epithelial cells [26]. This phenomenon may be explained by the fact that the external hydrophobic layer of the resting conidia is progressively lost during germination. Subsequently, the internal layer containing α- and β-glucans, chitin/chitosan and galactomannan is exposed on the conidial surface and can be recognized by the proper receptors on alveolar epithelial cells [27].

The proper phospho-cycling of cofilin plays an important role in efficient *A. fumigatus* internalization into A549 cells. We demonstrated that either silencing of host cell cofilin expression, elevating cofilin activity by overexpressing wild-type cofilin or expressing S3A cofilin (a non-phosphorylated form of cofilin) in epithelial cells impeded the invasion of *A. fumigatus* into A549 cells. Furthermore, the break in the status of cofilin phosphorylation by inhibiting the activity of LIMK or overexpressing SSH in A549 cells inhibited the internalization of *A. fumigatus*. These findings were in line with the previous report that *L. monogenes* internalization into Vero epithelial cells was controlled by the status of cofilin phosphorylation [28]. Cofilin has been reported to promote actin assembly or disassembly depending upon the concentration of cofilin relative to actin [6]. Thus, it can be deduced that the proper ratio of cofilin and p-cofilin is important for the efficient internalization of *A. fumigatus* into A549 cells. Dephosphorylation of p-cofilin by overexpressing SSH (which leads to an increase in cofilin) hindered the internalization. LIMK acts to integrate signals from a number of upstream pathways that regulate cofilin and the actin cytoskeleton, including the RhoA-ROCK pathway. Zeidan and Karmazyn reported that prevention of RhoA-ROCK-LIMK-cofilin-controlled actin polymerization mediated the anti-hypertrophic effect of adenosine receptor agonists in angiotensin II- and endothelin-1-treated cardiomyocytes [29]. Wang and colleagues also showed the important role of the Rho-ROCK-LIMK-cofilin pathway in TGF-β1-induced proliferation and cytoskeleton rearrangement in human periodontal ligament cells [30]. In line with these studies, we presented here that the internalization of *A. fumigatus* into A549 cells and the level of p-cofilin were also controlled by ROCK and RhoA. However, direct evidence for the concrete involvement of cofilin in RhoA/ROCK-controlled internalization should be provided in a future study.

Because cofilin phosphorylation induced by *A. fumigatus* is dependent on conidia germination and exposure of the conidial cell wall, we investigated the effect of β-1,3-glucan and its putative receptor dectin-1 on cofilin phosphorylation using two methods. First, we used β-1,3-glucan to stimulate the host cells, but did not find any significant alterations in cofilin phosphorylation. Second, we blocked the dectin-1 receptor with its antibody, but still did not detect a change in cofilin phosphorylation. These two results indicated that β-1,3-glucan signalling might not be associated with cofilin phosphorylation during *A. fumigatus* internalization into AECs.

Because *A. fumigatus* conidia may activate PLD via dectin-1 during their internalization into A549 cells [16], other receptors or signalling pathways might mediate cofilin phosphorylation; moreover, these two pathways might coordinate together to regulate *A. fumigatus* internalization. For instance, EGFR (epidermal growth factor receptor) is a critical receptor on epithelial cells that has been reported to be involved in the uptake of several pathogens into epithelial cells [31, 32]. Therefore, it would be interesting to test the role of EGFR or other possible receptors in *A. fumigatus* entry into A549 cells in the future. PLD inhibition was demonstrated to reduce *A. fumigatus* internalization [16]. Therefore, the cofilin-PLD correlation during *A. fumigatus* internalization is also worth further investigation because PLD might serve as the down-stream effector of p-cofilin in the acetylcholine receptor signalling pathway [33].

*A. fumigatus* swollen conidia can induce the release of proinflammatory cytokines (*i.e.*, IL-6, IL-8 and MCP-1) from A549 cells to promote the recruitment of inflammatory cells that may help protect against the conidia [26, 34]. Furthermore, Yang and colleagues proved that stimulation with cannabinoid Δ9-tetrahydrocannabinol (THC) induced the reduction of proinflammatory cytokine release by inhibiting cofilin expression in the MG-63 cell osteoarthritis model stimulated by LPS; in turn, cofilin overexpression abolished THC-induced anti-inflammation [35]. Therefore, it will be interesting to clarify the role of cofilin on *A. fumigatus-*induced expression of proinflammatory cytokines in A549 cells.

## Conclusions

In summary, these results indicated that *A. fumigatus* could induce cofilin phosphorylation in A549 cells during the early stage of internalization. The appropriate phosphorylation status of cofilin in A549 cells was essential for the effective internalization of swollen conidia. The RhoA-ROCK-LIM kinase pathway was involved in this process by decreasing the p-cofilin level in A549 cells.

## Abbreviations

AECs: Alveolar epithelial cells
ADF: Actin depolymerizing factor
F-actin: Actin filaments
LIMK: LIM kinases
SSH: Slingshot
PAK: p21-activated kinase
RhoA: Ras homologue gene family member A
ROCK: Rho-associated coiled-coil-containing kinase
HIV: Human immunodeficiency virus
ATCC: America Type Culture Collection
PBST: Phosphate-buffered saline supplemented with 0.1 % Tween-20
DMSO: Dimethyl sulfoxide
MOI: Multiplicity of infection
BSA: Bovine serum albumin
DAPI: 4’,6’ diamidino-2-phenylindole
HSV-1: Herpes simplex virus 1
p-cofilin: phosphorylated-cofilin
mpi: minute post infection
siRNA: small interfering RNA
cofilin WT: Wild-type cofilin
EGFR: Epidermal Growth Factor Receptor
